# Myocardial Lipin 1 knockout in mice approximates cardiac effects of human *LPIN1* mutations

**DOI:** 10.1172/jci.insight.134340

**Published:** 2021-05-10

**Authors:** Kari T. Chambers, Michael A. Cooper, Alison R. Swearingen, Rita T. Brookheart, George G. Schweitzer, Carla J. Weinheimer, Attila Kovacs, Timothy R. Koves, Deborah M. Muoio, Kyle S. McCommis, Brian N. Finck

**Affiliations:** 1 Division of Geriatrics and Nutritional Science, Department of Medicine, Washington University School of Medicine, St. Louis, Missouri, USA.; 2 Duke Molecular Physiology Institute and Sarah W. Stedman Nutrition and Metabolism Center, Departments of Medicine and Pharmacology and Cancer Biology, Duke University, Durham, North Carolina, USA.; 3 Department of Biochemistry & Molecular Biology, Saint Louis University School of Medicine, St. Louis, Missouri, USA.

**Keywords:** Cardiology, Metabolism, Cardiovascular disease, Intermediary metabolism, Mitochondria

## Abstract

Lipin 1 is a bifunctional protein that is a transcriptional regulator and has phosphatidic acid (PA) phosphohydrolase activity, which dephosphorylates PA to generate diacylglycerol. Human lipin 1 mutations lead to episodic rhabdomyolysis, and some affected patients exhibit cardiac abnormalities, including exercise-induced cardiac dysfunction and cardiac triglyceride accumulation. Furthermore, lipin 1 expression is deactivated in failing heart, but the effects of lipin 1 deactivation in myocardium are incompletely understood. We generated mice with cardiac-specific lipin 1 KO (cs-*Lpin1*^–/–^) to examine the intrinsic effects of lipin 1 in the myocardium. Cs-*Lpin1*^–/–^ mice had normal systolic cardiac function but mild cardiac hypertrophy. Compared with littermate control mice, PA content was higher in cs-*Lpin1*^–/–^ hearts, which also had an unexpected increase in diacylglycerol and triglyceride content. Cs-*Lpin1*^–/–^ mice exhibited diminished cardiac cardiolipin content and impaired mitochondrial respiration rates when provided with pyruvate or succinate as metabolic substrates. After transverse aortic constriction–induced pressure overload, loss of lipin 1 did not exacerbate cardiac hypertrophy or dysfunction. However, loss of lipin 1 dampened the cardiac ionotropic response to dobutamine and exercise endurance in association with reduced protein kinase A signaling. These data suggest that loss of lipin 1 impairs cardiac functional reserve, likely due to effects on glycerolipid homeostasis, mitochondrial function, and protein kinase A signaling.

## Graphical abstract

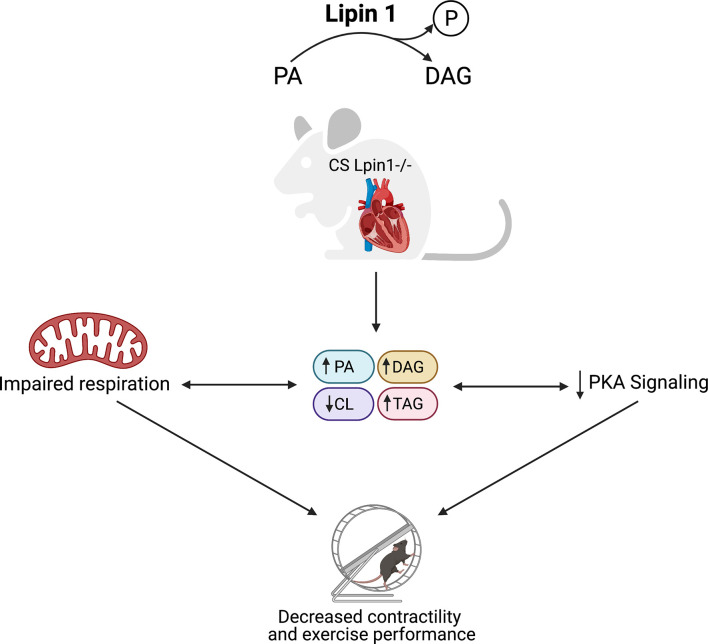

## Introduction

**Figure 1:**
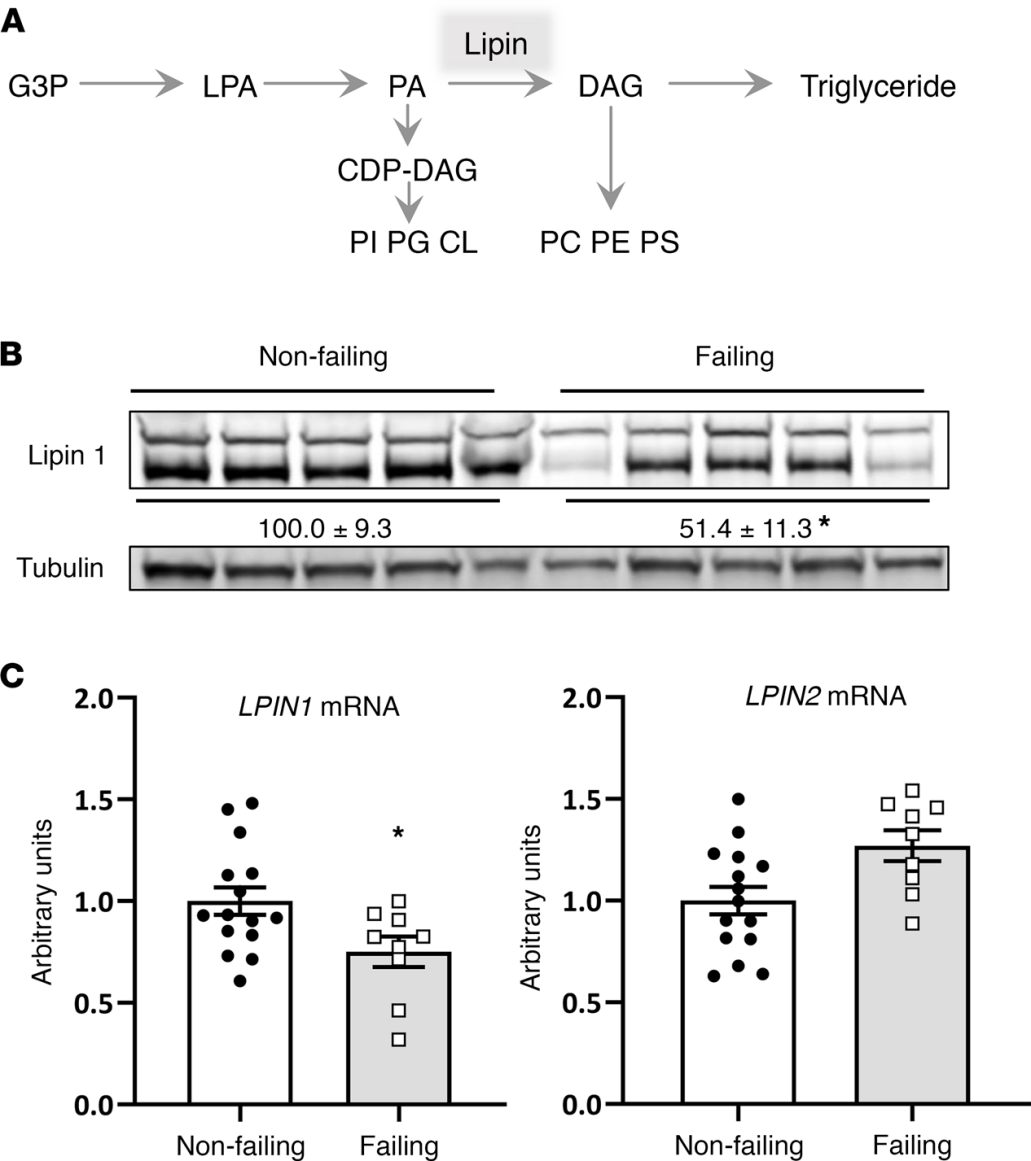
Lipin 1 is deactivated in failing human heart. (**A**) The schematic depicts the sequential acylation of glycerol-3-phosphate (G3P) to generate triglyceride. LPA, lysophosphatidic acid; PA, phosphatidic acid; DAG, diacylglycerol; CDP, cytosine diphosphate; PI, phosphatidylinositol; PG, phosphatidylglycerol; CL, cardiolipin; PC, phosphatidylcholine; PE, phosphatidylethanolamine; PS, phosphatidylserine. (**B** and **C**) Lipin 1 protein abundance (**B**) and mRNA expression (**C**) is significantly diminished in cardiac tissue of people with heart failure. Densitometry for the sum of both lipin 1 bands (normalized arbitrary units) is inset between the blots. Data represent mean ± SEM. *P < 0.05 by *t* test (*n* = 15 and 9/group).

Lipin 1 is a bifunctional protein that regulates metabolism by interacting with DNA-bound transcription factors (1) and also exhibiting Mg^2+^-dependent phosphatidic acid phosphohydrolase (PAP) activity (2). At the endoplasmic reticulum membrane, PAP enzymes dephosphorylate phosphatidic acid (PA) to form diacylglycerol (DAG) (Figure 1A), which is the penultimate step in triglyceride synthesis. In higher organisms, 3 lipin proteins (lipin 1, 2, and 3) are encoded by distinct genes. The lipin isoforms are differentially expressed in various tissues, with lipin 1 being the primary isoform in adipose tissue and in skeletal and cardiac muscle (3, 4).

In mice, mutations in lipin 1 result in neonatal hepatic steatosis and life-long lipodystrophy, insulin resistance, and progressive peripheral neuropathy (5, 6). However, mutations in the human gene encoding lipin 1 (*LPIN1*) do not result in a similar phenotype but have been linked to the development of recurrent, early-onset, pediatric rhabdomyolysis and myoglobinuria (OMIM #268200) (7–10). Rhabdomyolysis is an acute syndrome due to extensive injury of skeletal muscle resulting in the release of intracellular metabolites and proteins, including creatine kinase and myoglobin, into the systemic circulation. Untreated rhabdomyolysis can result in death from renal, cardiac, or hematologic dysfunction. Several deaths of *LPIN1* patients have been reported during rhabdomyolytic episodes, likely due to cardiac events (11). Work conducted with *fld* (12) and skeletal muscle–specific *Lpin1*-KO (13, 14) mice has revealed that loss of lipin 1 in muscle of mice leads to active and ongoing myopathy that is secondary to impairments in autophagy. This phenotypic manifestation diverges from the acute and nonprogressive nature of the human mutation, but it could be mechanistically linked.

*Lpin1* is well expressed in the myocardium and may play a role in this metabolically active organ. Previous work has shown that lipodystrophic, lipin 1–deficient *fld* mice exhibit cardiac dysfunction, though this was attributed to extracardiac factors and secondary to systemic alterations in metabolism (3). Recently, Legendre and colleagues examined cardiac function in 8 human patients with *LPIN1* mutations and found that cardiac function was normal in all but 1 of the patients at rest, but 3 of the subjects exhibited abnormal hemodynamics during exercise (15). The reasons for this impaired hemodynamic response were not definitively determined, but they may be related to energetic defects. *LPIN1* patients also exhibited accumulation of neutral lipid within the myocardium (15), and cardiac lipotoxicity has been implicated in the development of cardiac dysfunction in a variety of models (16, 17).

In this study, we show that cardiac lipin 1 expression is downregulated in human heart failure specimens. We also examined the role of lipin 1 in cardiac function, signaling, and metabolism by generating mice with cardiac-specific deletion of lipin 1. This approach was chosen in order to avoid the systemic effects of lipin deficiency on metabolic homeostasis. We found that, like most *LPIN1* patients, cardiac-specific loss of *Lpin1* in mice (cs-*Lpin1*^–/–^ mice) did not result in overt cardiac dysfunction. However, cs-*Lpin1*^–/–^ mice exhibited cardiac lipid accumulation and a blunted ionotropic response to dobutamine. Loss of lipin 1 led to impaired maximal mitochondrial respiration, reduced cardiolipin (CL) abundance, and attenuated protein kinase A (PKA) signaling. These data unveil potentially novel, cardiac-specific roles for lipin 1 in regulating cardiac lipid metabolism and contractile function in response to hemodynamic stress.

## Results

### The expression of LPIN1 is deactivated in human heart failure.

The protein abundance of lipin 1 and expression of *LPIN1* and *LPIN2* mRNA were quantified in heart samples from humans with heart failure, obtained at the time of left ventricular assist device (LVAD) implantation. Failing heart tissue was compared with cardiac tissue from donor hearts that were nonfailing but deemed unsuitable for transplant. Immunoblots using human heart lysates and an antibody against lipin 1 detected a doublet at approximately 130 kDa (Figure 1B; see complete unedited blots in the supplemental material; supplemental material available online with this article; https://doi.org/10.1172/jci.insight.134340DS1). This is often observed, and this doublet is due to the phosphorylated and dephosphorylated forms of the protein (18). We have shown that mutations in lipin 1 lead to a loss of both bands in human skeletal muscle lysates (9). We found that the protein abundance of both lipin 1 bands was decreased by nearly 50% in failing hearts compared with normal functioning controls (Figure 1B). In addition, the mRNA encoding lipin 1 (*LPIN1*) was also reduced in failing hearts compared with controls, whereas the expression of *LPIN2* tended to be increased (Figure 1C). However, it should be noted that, based on the Ct values obtained, *LPIN2* is expressed at much lower levels than *LPIN1* in myocardium (data not shown), consistent with low expression of this gene in heart (4).

### Generation of cardiac-specific Lpin1-KO mice.

**Figure 2:**
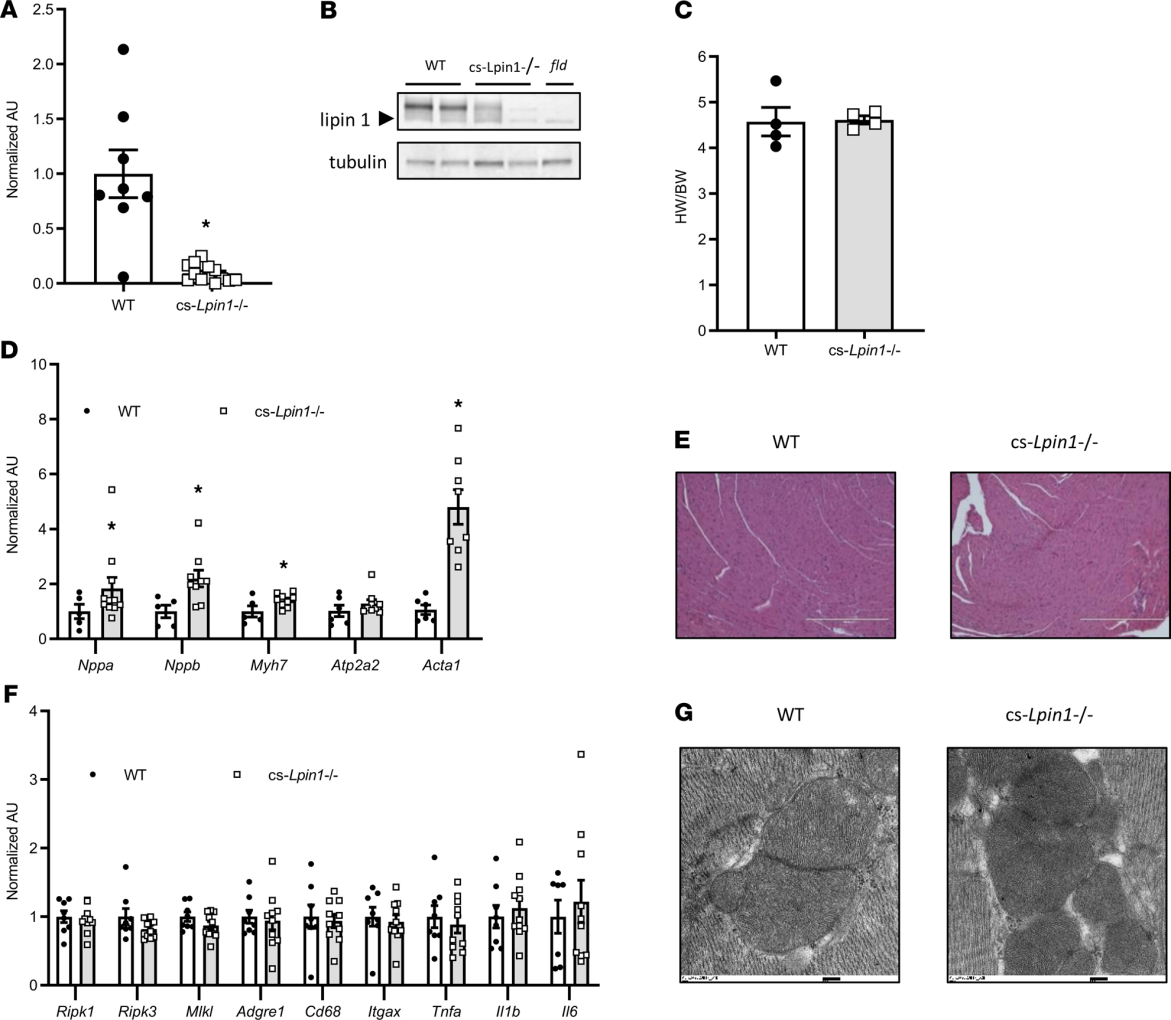
cs-*Lpin1^–/–^* mice are viable and exhibit minimal cardiac phenotype at baseline. (**A** and **B**) Lipin 1 mRNA expression (**A**) and protein abundance (**B**) is significantly diminished in the hearts of cs-*Lpin1^–/–^* mice. (**C**) No difference was observed in biventricular weight between cs-*Lpin1^–/–^* mice and littermate controls. (**D**) cs-*Lpin1^–/–^* mice had increased expression of fetal program genes (Nppa, Nppb, Myh7, and Acta1). (**E**) Histologic findings were normal in the hearts of 10-week-old cs-*Lpin1^–/–^* mice. Scale bars: 1000 μm. (**F**) No differences in gene markers of autophagy or inflammation are seen in the hearts of cs-*Lpin1^–/–^* mice. (**G**) Electron micrographs reveal no differences in mitochondrial ultrastructure in the hearts of cs-*Lpin1^–/–^* mice. Scale bars: 200 nm. Data represent mean ± SEM. *P < 0.05 by ANOVA (*n* = 4–9/group).

To characterize the effects of lipin 1 in the heart and avoid the systemic metabolic abnormalities present in mice with constitutive lipin 1 deficiency, we generated cs-*Lpin1*^–/–^ mice using recently described *Lpin1*-floxed mice (14) and mice expressing Cre under the control of the cardiac myosin light chain 2v promoter. Homozygous cs-*Lpin1*^–/–^ mice were viable and outwardly normal. Quantification of *Lpin1* mRNA expression showed a dramatic decrease in expression of this gene in cs-*Lpin1*^–/–^ heart compared with littermate controls not expressing Cre (WT; Figure 2A). Cardiac expression of lipin 1 protein was also markedly diminished in the KO mice compared with floxed controls not expressing Cre (denoted as WT) (Figure 2B). However, there was residual expression of lipin 1 protein in some hearts, likely due to expression in cells other than cardiac myocytes or incomplete recombination at the time point examined. Protein lysates from hearts of mice constitutively lacking lipin 1 (*fld*) showed complete loss of lipin 1 protein (Figure 2B).

### Cardiac lipin 1 deficiency does not lead to contractile dysfunction or myocyte death.

At sacrifice, biventricular weights were measured, and it was found that the biventricular/body mass ratio was not affected (Figure 2C). However, a significant increase in the cardiac expression of the fetal gene markers of hypertrophy (*Nppa, Nppb, Myh7*, and *Acta1*) in cs-*Lpin1*^–/–^ mice compared with littermate controls was identified (Figure 2D). Echocardiographic characterization of cardiac mass and function revealed no evidence of cardiac hypertrophy in 10-week-old male or female cs-*Lpin1*^–/–^ mice compared with littermate controls, nor were any deficits in systolic function detected (Table 1).

We have recently shown that deletion of lipin 1 in skeletal muscle of mice impairs autophagy and accumulation of abnormal mitochondria, and it produces active myopathy, including loss of skeletal myocytes to necrotic cell death, macrophage infiltration, and sterile inflammation of muscle (14). Histologic evaluation of cardiac sections from cs-*Lpin1*^–/–^ mice found no evidence of these myopathic changes (Figure 2E), nor was the expression of gene markers for these processes elevated (Figure 2F). Electron microscopy of cardiac mitochondria from cs-*Lpin1*^–/–^ mice showed no ultrastructural abnormalities of mitochondria (Figure 2G).

### Cardiac lipid accumulation in hearts of cs-Lpin1^–/–^ mice.

**Figure 3:**
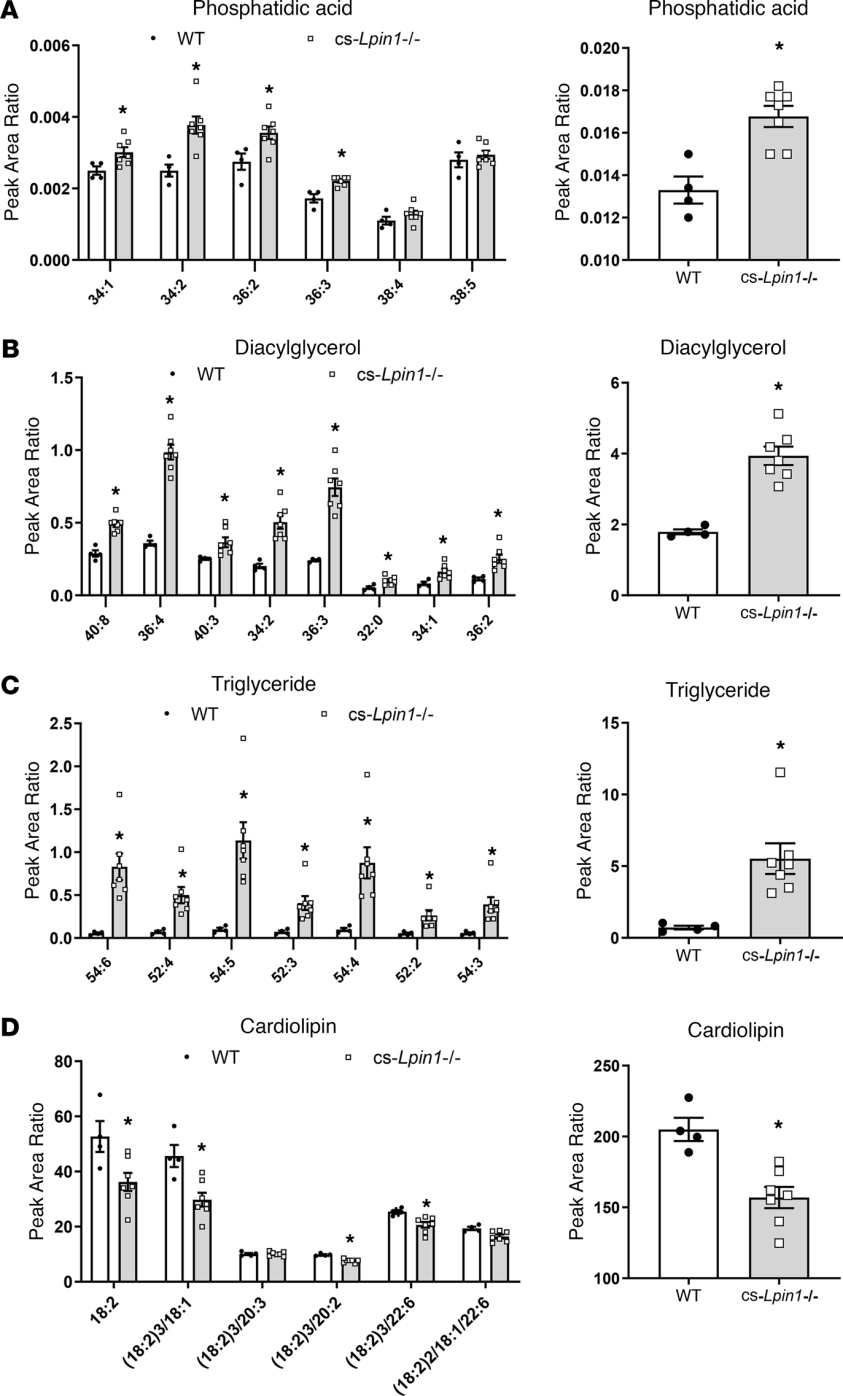
cs-*Lpin1*^–/–^ mice exhibit cardiac lipid accumulation. (**A**–**C**) cs-*Lpin1*^–/–^ mice have elevated levels of phosphatidic acid (**A**), diacylglycerol (**B**), and triacylglycerol (**C**) in their hearts compared with littermate controls. (**D**) Mass spectroscopy analysis displays reduced levels of cardiolipin in the hearts of cs-*Lpin1*^–/–^ mice. Data represent mean ± SEM. *P < 0.05 by ANOVA (*n* = 4–6/group).

Recent work conducted using ^1^H-MRS has suggested that *LPIN1* patients exhibit accumulation of cardiac triglyceride (15). We used mass spectrometry to quantify cardiac PA, DAG, and triglyceride species. We found that several species of PA and total PA abundance was increased in hearts of cs-*Lpin1*^–/–^ mice compared with littermate controls (Figure 3A). The abundance of DAG and triglyceride, including most of the major species measured (Figure 3, B and C), was counterintuitively increased in hearts of cs-*Lpin1*^–/–^ mice. However, this is consistent with recent results obtained in skeletal muscle-specific *Lpin1*^–/–^ mice (13, 14) and human *LPIN1* patients (15). Interestingly, the abundance of CL, a lipid that plays an important role in regulating mitochondrial oxidative metabolism, was decreased in cs-*Lpin1*^–/–^ mice compared with WT controls (Figure 3D). These data show that cardiac-specific lipin 1 deletion leads to an alteration in the abundance of several lipid species in the heart.

### Diminished mitochondrial respiration in hearts of cs-Lpin1^–/–^ mice.

**Figure 4:**
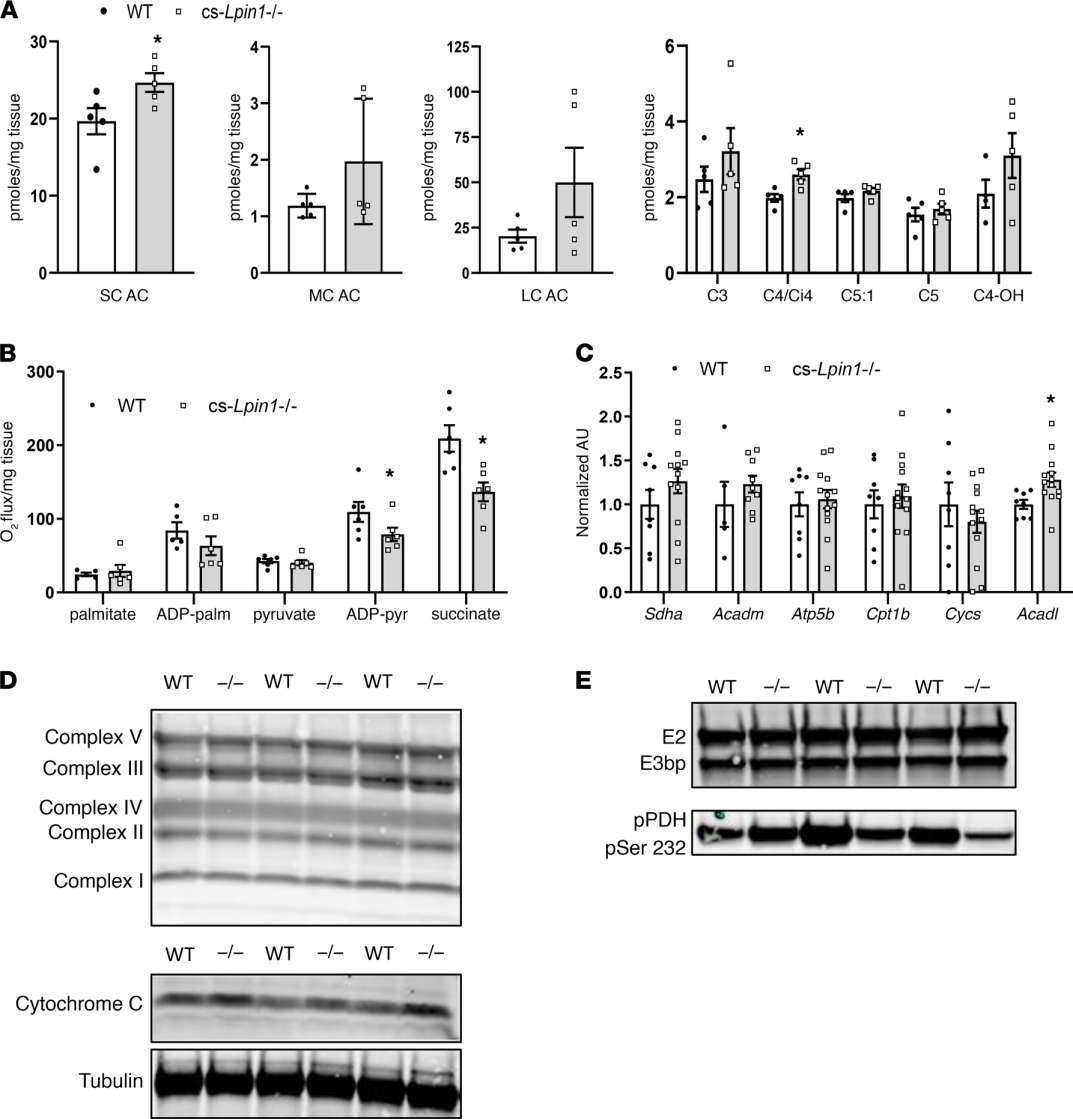
cs-*Lpin1*^–/–^ hearts have decreased levels of ADP-stimulated mitochondrial respiration. (**A**) Metabolomic analysis revealed accumulation of short-chain acylcarnitines. (**B**) Mitochondrial respiration studies reveal a decreased response in cs-*Lpin1*^–/–^ hearts in response to ADP-succinate–stimulated respiration. (**C**) qPCR of analysis of cs-*Lpin1*^–/–^ and WT cardiac muscle. (**D**) Western blot analysis showing protein expression of mitochondrial complexes (I–IV) and cytochrome C. (**E**) Western blot analysis of pyruvate dehydrogenase complex and phosphorylation of PDH in cardiac muscle of WT and cs-*Lpin1*^–/–^ mice. Data represent mean ± SEM. *P < 0.05 by *t* test (*n* = 4–7/group).

Diminished CL and accumulation of lipid in the myocardium with lipin 1 deficiency could suggest impairments in mitochondrial oxidative function. Consistent with this, metabolomic analyses of myocardium after an overnight fast revealed a significant accumulation of short-chain acyl-carnitines (SCAC) (containing 2–6 carbons) with a specific increase in C4 and Ci4 metabolites in *cs-Lipin1^–/–^* mice compared with WT controls (Figure 4A). The abundance of medium-chain AC (MCAC) and long-chain AC (LCAC) tended to be increased but was not significantly greater. The abundance of organic acids and amino acids was largely unaffected (Supplemental Table 1).

To evaluate mitochondrial function, LV cardiac tissue fibers were permeabilized, and mitochondrial respiration was assessed. Palmitoylcarnitine-mediated respiration was unaltered by the loss of cardiac lipin 1 (Figure 4B). In contrast, maximal pyruvate-mediated respiration and succinate-mediated respiration was lower in permeabilized strips from *cs-Lipin1^–/–^* mice (Figure 4B). Loss of lipin 1 did not affect the mRNA expression (Figure 4C) or protein abundance (Figure 4D) of components of the electron transport chain or cytochrome C. We also did not detect reduced expression of the components of the pyruvate dehydrogenase (PDH) complex or increased inhibitory phosphorylation of the PDH at Ser232 (Figure 4E). Collectively, these data suggest that loss of lipin 1 in the myocardium leads to reduced maximal respiration with pyruvate or succinate in association with reduced CL abundance.

### Loss of lipin 1 does not affect the response to transverse aortic constriction (TAC).

To determine whether lipin deficiency caused the heart to be more sensitive to pathologic stimuli, we subjected WT and cs-*Lpin1*^–/–^ mice to pressure overload by the TAC surgical model. Deletion of lipin 1 in the heart did not affect the hypertrophic response to TAC, as evidenced by an equivalent increase in LV wall thickness in systole (LVPWs) and diastole (LVPWd) and an increase in the LV mass index (LVMI) (Table 2). Additionally, contractile function was unaltered by TAC as compared with WT mice in *cs-Lipin^–/–^* mice (Table 2).

### Impaired ionotropic response and PKA signaling in cs-Lpin1^–/–^ mice.

**Figure 5:**
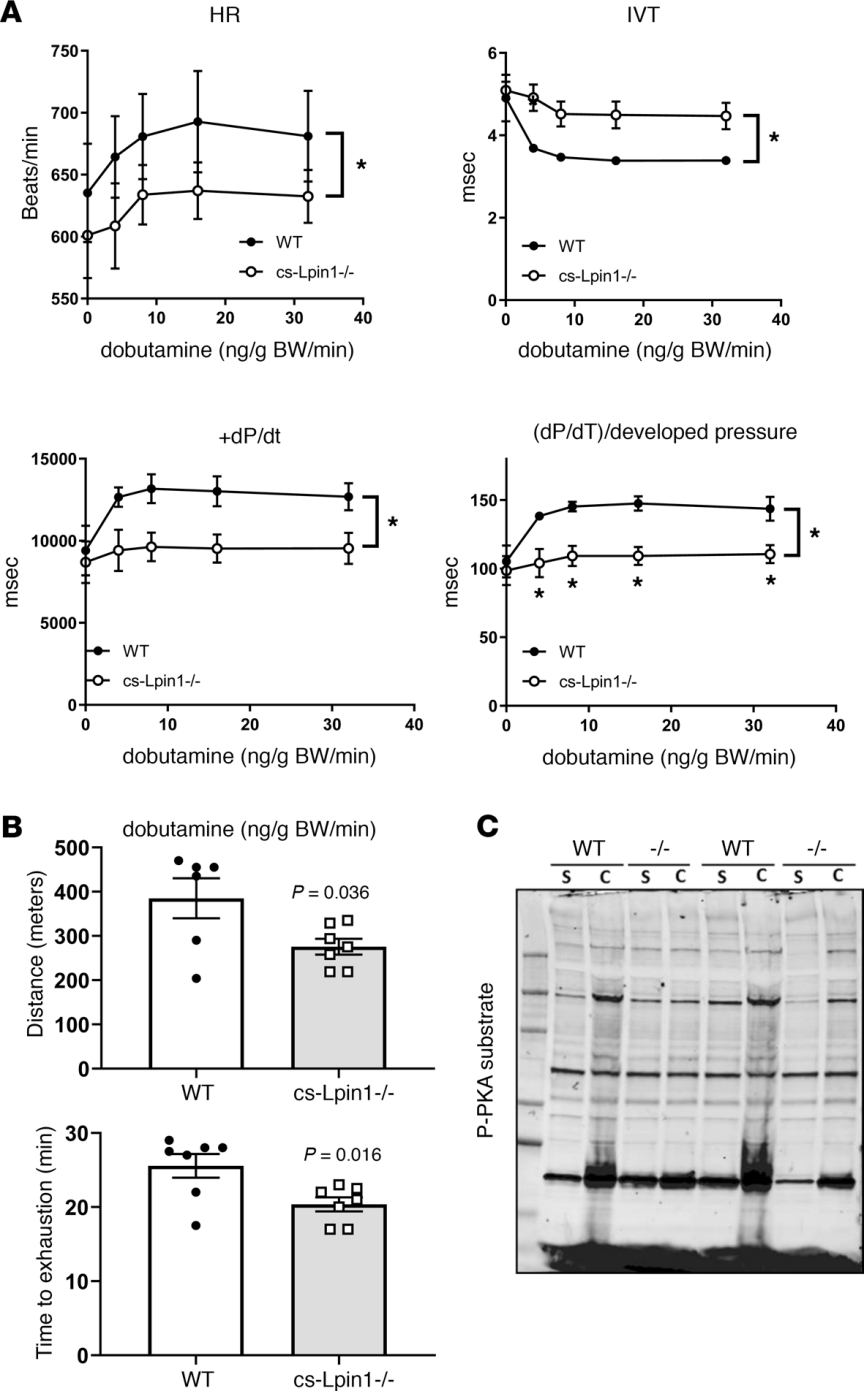
cs-*Lpin1^–/–^* mice exhibit reduced response to β-agonist stimulation. (**A**) Dobutamine stimulation during cardiac catheterization to measure HR, IVT, +dP/dt, and (dP/dt)/developed pressure. Data represent mean ± SD. *P < 0.05 by repeated-measures ANOVA (*n* = 4–6/group). (**B**) Graphs depict distance run and time to exhaustion during forced treadmill exercise studies (*n* = 7 per group). *P < 0.05 by *t* test. (**C**) Western blot analysis of P-PKA substrates in response to clenbuterol stimulation.

We also assessed contractile reserve in cs-*Lpin1*^–/–^ mice by closed chest cardiac catheterization studies. Hemodynamic measurements were obtained in response to increasing doses of dobutamine. During the course of the dobutamine infusion, heart rate was lower in cs-*Lpin1*^–/–^ mice versus WT controls (Figure 5A). Isovolumic relaxation time (IVT), which typically declines with dobutamine, was higher in cs-*Lpin1*^–/–^ mice compared with WT controls. Lipin 1 deficiency did not impact the effects of dobutamine on ejection time, developed pressure, LV end-diastolic pressure, or negative dP/dt (Figure 5A). However, +dP/dt and the ratio of +dP/dt to developed pressure increased in WT mice but was lower in cs-*Lpin1*^–/–^ mice in response to increasing levels of dobutamine (Figure 5A). Collectively, these data suggest that loss of lipin 1 leads to diminished cardiac reserve.

To test whether this was associated with functional effects on exercise endurance, which may be reduced in some patients with LPIN1 deficiency, WT and cs-*Lpin1*^–/–^ mice underwent a graded intensity exercise bout on a motorized treadmill. We found that cs-*Lpin1*^–/–^ mice ran for less time and distance compared with WT littermate control mice (Figure 5B). This suggests that cardiac intrinsic effects of lipin 1 deficiency leads to reduced exercise capacity.

We have previously shown that lipin 1 deficiency in adipose tissue or liver leads to impaired PKA signaling due to activation of phosphodiesterase 4 in response to PA accumulation (19). Consistent with this, when cs-*Lpin1*^–/–^ mice and littermate controls were injected with the β2 adrenergic agonist, clenbuterol, we found decreased phosphorylation of several PKA targets in the hearts of cs-*Lpin1*^–/–^ mice compared with littermate mice, as assessed by a phospho-PKA substrate antibody (Figure 5C).

## Discussion

Lipin 1 plays an important role in intermediary metabolism, but the effects of lipin 1 on cardiac metabolism and function remain poorly understood. Recent work conducted in patients with mutations in the gene encoding lipin 1 has suggested that some patients develop cardiac dysfunction and hemodynamic abnormalities when challenged with an increased workload (15). Magnetic resonance spectroscopic analyses of these hearts showed a paradoxical increase in cardiac triglyceride content. Herein, we show that mice with cardiac restricted lipin 1–KO show a similar cardiac phenotype. Specifically, cardiac function is relatively normal at baseline, but the mice exhibit impaired contractility and cardiac reserve when challenged with dobutamine. Myocardial accumulation of several lipids, including PA, DAG, and triglyceride, was also noted. These mice can serve as a model for studying the cardiac effect of lipin 1 deficiency to further define mechanism and explore potential treatments.

Previous work on the effects of lipin 1 on cardiac metabolism has employed *fld* mice, a model with spontaneous deletion of lipin 1 (3). From these studies, it is clear that lipin 1 is the primary PAP enzyme in the heart (3, 4). While these studies have provided useful information, the interpretation of the findings was complicated by the complex metabolic phenotype of *fld* mice, which includes severe lipodystrophy and metabolic abnormalities (6, 20); this led us to develop a cardiac-specific model. At 10 weeks of age, *fld* mice exhibited reduced fractional shortening (3), whereas cardiac-specific deletion of lipin 1 did not lead to overt systolic or diastolic function at baseline. One interpretation of these results is that the systemic metabolic abnormalities of *fld* mice impacted cardiac function. Indeed, cardiac dysfunction in *fld* mice was observed in vivo but not in vitro (3). However, it is not clear whether this relates well to the cardiac abnormalities in patients with *LPIN1* mutations, since these patients are not lipodystrophic.

As in human patients with *LPIN1* mutations (3), we observed cardiac DAG and triglyceride accumulation in lipin 1–deficient hearts. There are multiple lipin 1–independent pathways to synthesize DAG from phospholipid that could explain this phenomenon, and this observation fits with the emerging concept that, in many tissues (9, 13, 14), lipin 1 is not required for normal rates of triglyceride synthesis. It is possible that lipin 1 PAP activity, instead, plays an important signaling role by regulating the abundance of PA or other lipids. Indeed, a consistent observation in mice with KO of lipin 1 is an attenuation of PKA signaling. Adipocyte-specific and liver-specific lipin 1–KO mice exhibit impaired PKA signaling (19), and this was also observed in the heart in the present study and by Kok and colleagues in *fld* mice (3). Mechanistically, PA accumulation due to lipin 1 deficiency activates phosphodiesterase 4, which degrades cAMP to attenuate PKA signaling (19). Consistent with a decline in PKA activity, *fld* mice are also reported to have reduced phosphorylation of hormone sensitive lipase, an exemplar PKA target, in heart (3) and adipose tissue (19). Alterations in PKA-stimulated lipolysis through this pathway could also contribute to the accumulation of DAG and triglyceride in cs-*Lpin1*^–/–^ hearts by reducing turnover of these lipids.

Also consistent with impaired PKA signaling, cardiac reserve was reduced in the KO mice during inotropic stimulation. It is also possible that the attenuated response to dobutamine could be energetic in nature, since mitochondrial respiration was impaired in mice with cardiac lipin 1 KO. We did not detect any abnormalities in mitochondrial mass or protein abundance, which is different than skeletal muscle of lipin 1–deficient mice (14), but we did find that the abundance of CL, a phospholipid found in the inner mitochondrial membrane, was reduced in lipin 1–deficient hearts. CL is derived from PA but does not require PAP activity to be synthesized (Figure 1A). Deficiency in CL leads to Barth syndrome, which is associated with cardiac defects due to mitochondrial abnormalities (21). Nonetheless, these mitochondrial abnormalities were much less severe than the mitochondrial phenotype observed in skeletal muscle (14). The myopathy observed in lipin 1–deficient skeletal myocytes was also not observed in cardiac myocytes. The mechanistic explanation for the tissue-specific effects of lipin 1 deficiency on mitochondrial function and myocyte viability remains to be determined.

We have previously demonstrated that lipin 1 protein abundance is diminished in mouse models of heart failure coincident with accumulation of PA in the myocardium (4). Herein, we demonstrate that lipin 1 is also deactivated in failing human heart. Although there was insufficient sample mass to quantify PA or other lipids in these samples, it is likely that PA may be increased in these samples, as it is in failing mouse heart. It remains to be determined whether reduced lipin 1 activity and accumulation of PA affects cardiac contractility in failing heart. While the cs-*Lpin1*^–/–^ mice did not exhibit cardiac dysfunction after TAC surgery, it is probable that a lipin 1 gain-of-function approach might be the better way to test the role of lipin 1 in heart failure, since lipin 1 is deactivated by pathologic stimuli.

In conclusion, these studies define cardiac-specific roles for lipin 1 in regulating cardiac lipid levels and cardiac reserve in response to functionally demanding stimuli. However, mice with cardiac deletion of lipin 1 did not exhibit a propensity to develop heart failure after TAC — a pathologic stimulus that can cause mice with mild cardiomyopathy to develop overt dysfunction. These findings may have implications for the rare patients with *LPIN1* mutations, as well as general impact for metabolic adaptations that occur in failing heart. Future work will continue to examine the effects of lipin 1 deficiency on metabolism and function and will provide further insight into the molecular mechanisms involved.

## Methods

### Human heart samples.

Failing human LV tissue was obtained from the Washington University Translational Cardiovascular Tissue Core at the time of LVAD placement. Nonfailing control heart tissue was obtained from Mid-America Transplant (St. Louis, Missouri, USA) from hearts deemed unsuitable for transplantation due to donor age, coronary artery disease, or high-risk behavioral profile. The collected piece of cardiac tissue was trimmed, rinsed in saline, and then frozen in liquid nitrogen and stored at –80°C until analyzed.

### Generation of cs-Lpin1^–/–^ mice.

The generation of *Lpin1-*floxed mice was recently described (14). Homozygous *Lpin1*-floxed mice (B6[Cg]-*Lpin1*tm1c[EUCOMM]^Hmgu/FincJ^; The Jackson Laboratory, stock no. 032117) in the C57BL/6J background were crossed with mice expressing Cre recombinase under the endogenous myosin light chain 2v promoter (B6.129S4[Cg]-Myl2tm1[cre]^Krc/AchakJ^; The Jackson Laboratory, stock no. 029465) to homozygosity for the *Lpin1*-floxed allele. Littermates not expressing Cre (WT mice) were used as control mice in all experiments. *Fld* mice in the BALB/C background, used as a negative control in Figure 2B, were also obtained from The Jackson Laboratory (BALB/cByJ-*Lpin1^fld^*/J; stock no. 001592). Mice were studied at 10–16 weeks of age, and both sexes of mice were studied as indicated.

### Western blotting.

Protein from frozen heart tissue was homogenized in 1 mL ice-cold lysis buffer (20 mM Tris, 15 mM NaCl, 1 mM EDTA, 0.2% NP-40, 10% glycerol, supplemented with 1 mM activated Na_3_VO_4_, 1 mM phenylmethanesulfonyl fluoride, 5 mM sodium fluoride (all from MilliporeSigma), and 1× Complete protease inhibitor cocktail tablet; Roche) using high-speed tissue disruption with a stainless steel bead and the TissueLyser II (Qiagen). Lysates were then rotated at 4°C for 1 hour. Samples were then centrifuged (15,000*g* for 15 minutes at 4°C), and supernatants were transferred to a new microfuge tube. Protein quantification was performed using the bicinchoninic acid (BCA) assay according to the manufacturer’s protocol (Pierce Biotechnology). The remaining lysates were aliquoted (to prevent repeated freeze-thaw cycles) and stored at –80°C. Lysates were subjected to PAGE analysis using Criterion precast gels (Bio-Rad) and were then transferred to PVDF membranes. The blots were blocked in 5% BSA/1× TBST for 1 hour at room temperature and were incubated overnight (4°C with rocking) with the appropriate primary antibody: lipin 1 (sc-98450, Santa Cruz Biotechnology Inc.); OXPHOS (MS604-300, Abcam); PDH pSer232 (AP1063, MilliporeSigma); PDH (ab110416, Abcam); cytochrome C (ab110325, Abcam); phospho-PKA substrate (RRXS/T; 9624, Cell Signaling Technology), and tubulin (T5168, Sigma-Aldrich). Blots were then washed 3× in TBST and incubated with appropriate secondary antibodies: goat anti–rabbit IRDye 680 (catalog 926-68021) or goat anti–mouse IRDye 680 (catalog 926-32220) (both from Li-Cor Biosciences) for 1 hour at room temperature; they were washed again 3× in TBST and imaged with the Odyssey Imaging System (Li-Cor Biosciences).

### Quantitative PCR.

Total RNA was isolated from hearts using the RNAzol method (RNA-Bee; Tel-Test). Complementary DNA was synthesized by using a high-capacity reverse transcription kit (Applied BioSystems). Real-time PCR was performed using an ABI PRISM 7500 sequence detection system and a SYBR green master mix (Applied BioSystems). Primer sequences can be found in Supplemental Table 2. Arbitrary units of target cDNA were normalized to the levels of the housekeeping gene 36B4 cDNA. Oligonucleotide sequences are found in Supplemental Table 2.

### Lipid analysis.

The abundance of PA, DAG, triglyceride, and CL in mouse hearts was determined as described with modification (22). The LC-MS analysis was performed either with a Shimadzu 10A HPLC system and a Shimadzu SIL-20AC HT auto-sampler coupled to a Thermo Scientific TSQ Quantum Ultra triple quadrupole (TQ) mass spectrometer operated in selected reaction monitoring mode or with a Thermo Fisher Scientific Vantage TSQ mass spectrometer with Thermo Accela UPLC operated by Xcalibur software using selected ion monitoring mode. PA-(14:0)_2_, DAG-(15:0)_2_, triglyceride-(17:0)_3_, and CL-(14:0-14:0)_2_ were used as internal standards. Quantification of lipids was based on the ratio of the peak area of the analyte to the internal standard. For example, the ratio of CL-(18:2/18:2)_2_ and CL-(14:0-14:0)_2_ is used for measurement of CL-(18:2/18:2)_2_.

### Metabolomics analysis.

Mice used for targeted metabolomic analyses were fasted for 18 hours, deeply anesthetized with isoflurane inhalation, and euthanized by excision of the beating heart. Hearts were freeze clamped with frozen tongs, frozen in liquid nitrogen, and stored at –80°C until they were collectively processed and analyzed. Flash-frozen hearts were pulverized to a fine powder in a liquid nitrogen–chilled percussion mortar and pestle and were weighed into prechilled 2 mL tubes. A chilled 5 mm homogenizing bead was added to samples, and tissue was diluted to 50 mg/mL with 50% acetonitrile containing 0.3% formate, homogenized for 2 minutes at 30 Hz using a TissueLyser II (Qiagen), and aliquoted for metabolite assays. For all metabolite analyses, tissues and homogenates were kept on ice, centrifuged at 4°C, and — when ready to measure — were placed in an autosampler kept at 4°C.

Amino acids and acylcarnitines were analyzed by flow injection electrospray ionization tandem mass spectrometry and quantified by isotope or pseudoisotope dilution. Extracted heart samples were spiked with a cocktail of heavy-isotope internal standards (Cambridge Isotope Laboratories; or CDN Isotopes) and deproteinated with methanol. The methanol supernatants were dried and esterified with either acidified methanol or butanol for acylcarnitine or amino acid analysis, respectively. Mass spectra for acylcarnitine and amino acid esters were obtained using precursor ion and neutral loss scanning methods, respectively. The spectra were acquired in a multichannel analyzer (MCA) mode to improve the signal-to-noise ratio. The data were generated using a Waters TQ detector equipped with Acquity UPLC system and a data system controlled by MassLynx 4.1 operating system (Waters). Ion ratios of analyte to a respective internal standard computed from centroided spectra were converted to concentrations using calibrators constructed from authentic aliphatic acylcarnitines and amino acids (MilliporeSigma; Larodan), as well as Dialyzed Fetal Bovine Serum (MilliporeSigma).

Organic acids were analyzed by capillary gas chromatography/mass spectrometry (GC/MS) using isotope dilution techniques employing Trace Ultra GC coupled to ISQ MS operating under Xcalibur 2.2 (Thermo Fisher Scientific). The supernatants of tissue homogenates were spiked with a mixture of heavy isotope–labeled internal standards, and the keto acids were stabilized by ethoximation. The organic acids were acidified and extracted into ethyl acetate. The extracts were dried and derivatized with N,O-bis(Trimethylsilyl) trifluoroacetamide. The organic acids were quantified using ion ratios determined from single ion recordings of fragment ions, which are specific for a given analyte and its internal standard. These ratios were converted to concentrations using calibrators constructed from authentic organic acids (MilliporeSigma).

### Transverse aortic constriction.

Surgery was performed by the Mouse Cardiovascular Phenotyping Core (MCPC) at Washington University School of Medicine. In brief, mice were anesthetized with a mixture of xylazine (10 mg/kg) and ketamine (100 mg/kg), and the TAC procedure was performed by a surgeon blinded to the genotype of the mice as described (23). Following 2 weeks of pressure overload using TAC, the animals underwent 2-D echocardiographic imaging for LV structure and function. The day after Echo analysis, the mice were euthanized, and the hearts were excised for heart weight/body weight ratio; they were then were snap frozen for further analyses.

### Hemodynamics.

WT and cs-*Lpin1*^–/–^ mice underwent in vivo hemodynamic evaluations using a graded dobutamine infusion to assess cardiac reserve as described (24). These studies were also carried out by the MCPC. Briefly, mice were anesthetized with 2% isoflurane and were maintained on 1.5% isoflurane throughout the duration of the procedure. Closed chest cardiac catheterization was performed by identifying and cannulating the right carotid artery, and a 1.4 Fr Scisense catheter was advanced into the ascending aorta, and then retrograde across the valve into the left ventricle. After acquiring baseline measurements, dobutamine was serially infused at rates of 4, 8, 16, and 32 ng/g BW/min. Hemodynamic measurements were then recorded in real time and analyzed offline.

### Exercise protocol.

For exercise studies, male mice were run to exhaustion on a closed 6-lane treadmill (Columbus Instruments) equipped with a shock grid at the back of each belt that delivered a mild electrical stimulus to encourage continuous running. Food was removed 5 hours before exercise, water was available ad libitum, and bedding was replaced with Aspen chip bedding. Mice were acclimated to the treadmill with 0° incline at 0 m/min for 5 minutes. The speed was then increased to 5 m/min and maintained for 5 minutes. After 5 minutes of continuous running, the speed was increased so that, after the first 5 minutes, the speed reached 10 m/min, 5 minutes later the speed reached 15 m/min, and so on, until mice reached exhaustion. Speeds did not exceed 30 m/min. Exhaustion was determined by refusal of mice to remain on the treadmill belt for 10 seconds.

### Echocardiography.

Two-dimensional M-mode echocardiography was performed by the MCPC using a VEVO 2100 ultrasound machine. Mice were anesthetized with Avertin, which preserves the heart rate at physiologic levels throughout the imaging protocol. All echocardiography was performed by an investigator who was blinded to genotype.

### Mitochondrial respiration.

LV cardiac tissue was permeabilized with saponin (5 mg/mL), and high-resolution respirometry was conducted using an Oxygraph O2k (Oroboros Instruments). A total of 3–4 mg of permeabilized tissue was added to 2 mL of MiR05 (Oroboros) respiration buffer supplemented with creatine and blebbistatin. Respiratory substrates utilized were malate (2 mM), pyruvate (5 mM), palmitoyl-carnitine (10 mM), ADP (2 mM), succinate (10 mM), FCCP (0.5 μM, repeated injections until maximal respiration occurred), and rotenone (0.5 μM). All measures were normalized to tissue weight.

### Statistics.

Statistical comparisons were made using a 2-tailed Student’s *t* test or 2-way ANOVA with Tukey’s post hoc analysis performed where appropriate. All data, unless otherwise noted, are presented as mean ± SEM. *P* ≤ 0.05 WAS considered statistically significant.

### Study approval.

All animal experiments were approved by the IACUC of Washington University. Human heart samples were collected with written informed consent received from participants as part of an IRB-approved protocol at the Washington University School of Medicine.

## Author contributions

KTC and MAC contributed equally to this work, and their authorship order was determined alphabetically by last name. KTC designed and participated in all cs-*Lipin^–/–^* mouse experiments. MAC performed mitochondrial function and performed Western blots and RNA analyses. ARS performed experiments in cs-*Lpin1*^–/–^ mice and measured gene expression. RTB conducted treadmill exercise studies and edited the manuscript. GGS generated the cs-*Lpin1*^–/–^ mouse line. CJW performed both the TAC surgeries and the hemodynamic evaluations, and AK conducted echocardiographic analyses of all mice. TRK, DMM, and KSM assisted in data collection and analyses, as well as in writing and editing the manuscript. BNF wrote the manuscript and assisted in the design of all experiments. All authors were involved in writing and editing the manuscript.

## Supplementary Material

Supplemental data

Supplemental Table 1

Supplemental Table 2

## Figures and Tables

**Table 1:**
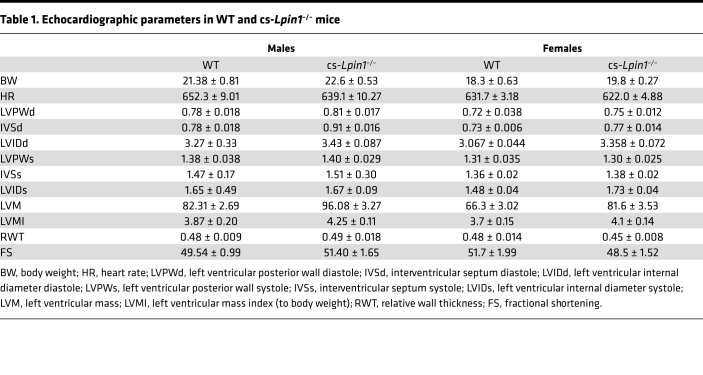
Echocardiographic parameters in WT and cs-*Lpin1*^–/–^ mice

**Table 2:**
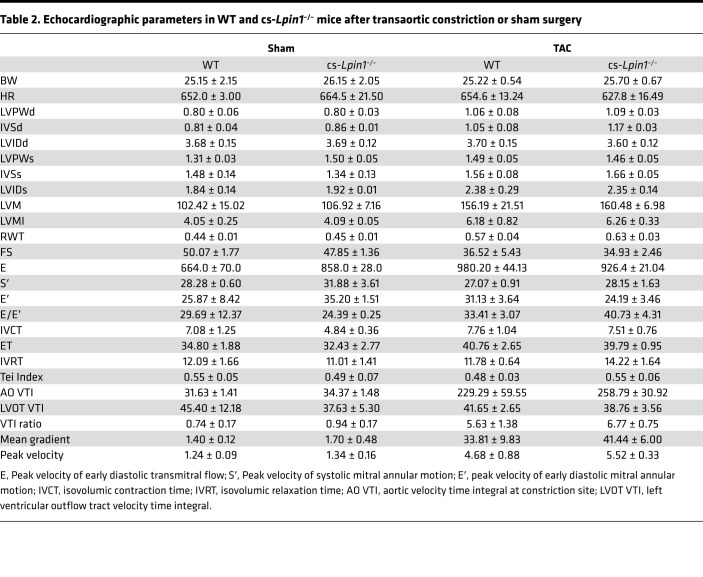
Echocardiographic parameters in WT and cs-*Lpin1*^–/–^ mice after transaortic constriction or sham surgery

## Version 1 05/10/2021

Electronic publication
