# Supplementary figures and images for: Myocardial Lipin 1 knockout in mice approximates cardiac effects of human LPIN1 mutations
Source: JCI Insight. 2021 May 10;6(9):e134340. doi: 10.1172/jci.insight.134340 (PMC8262319; doi:10.1172/jci.insight.134340)

Figure 1

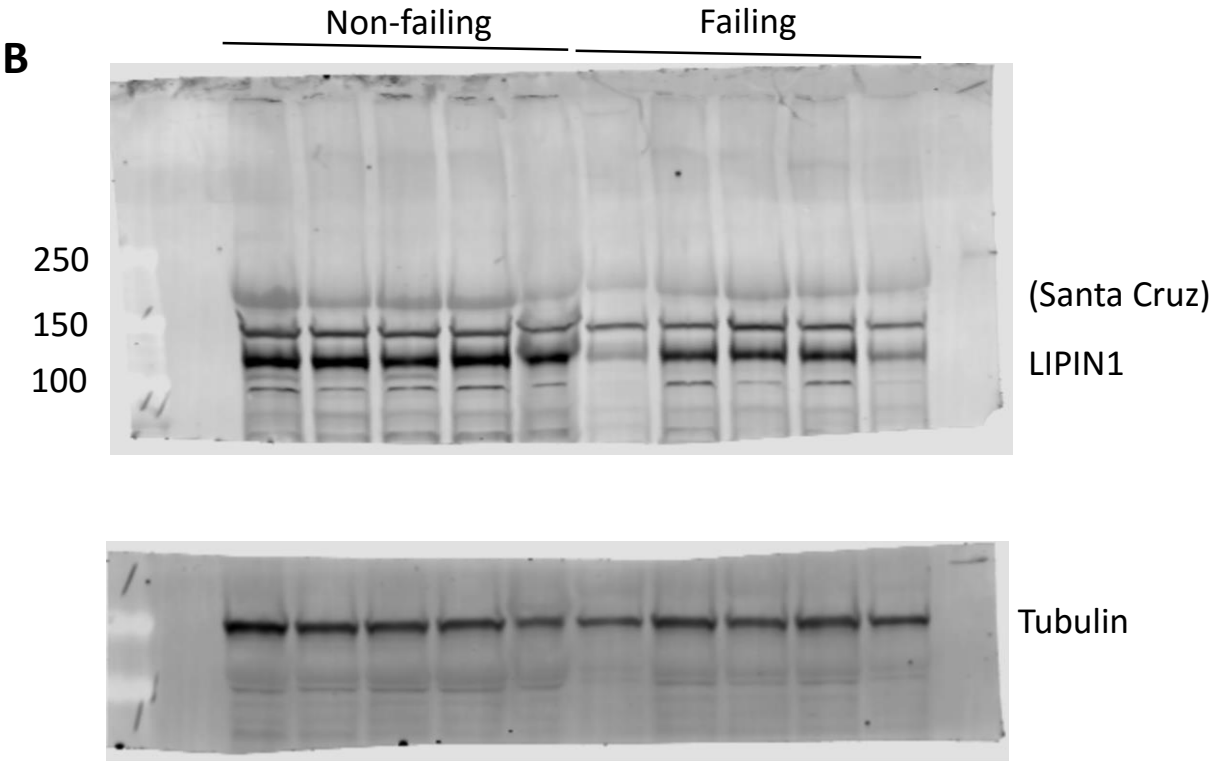

B

Lipin1

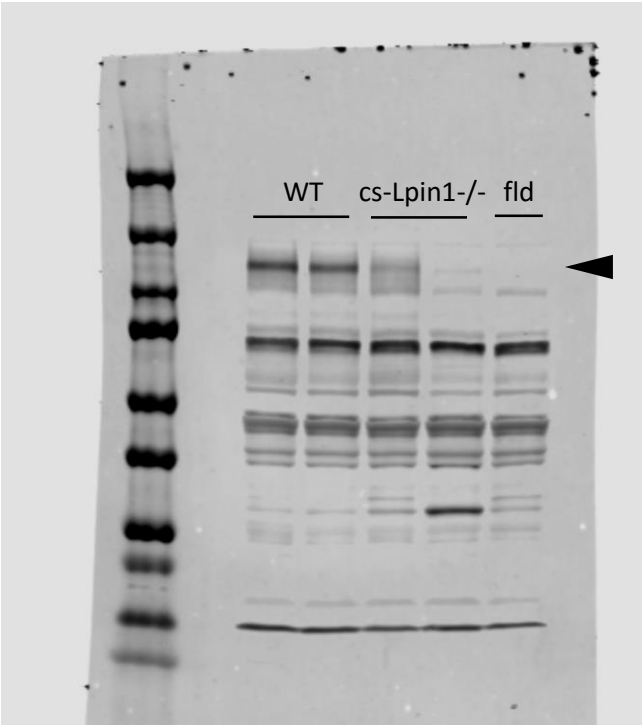

$\alpha$ Tubulin

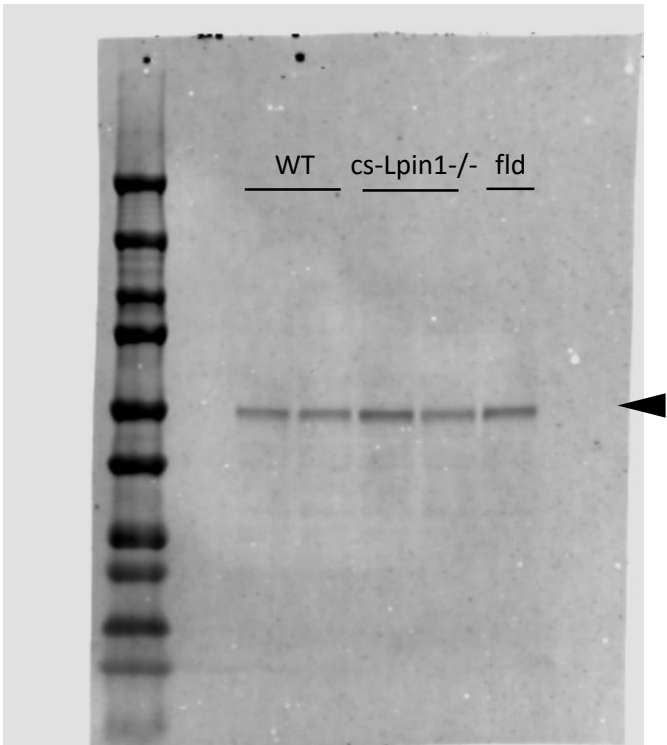

**Figure 4**

**D**

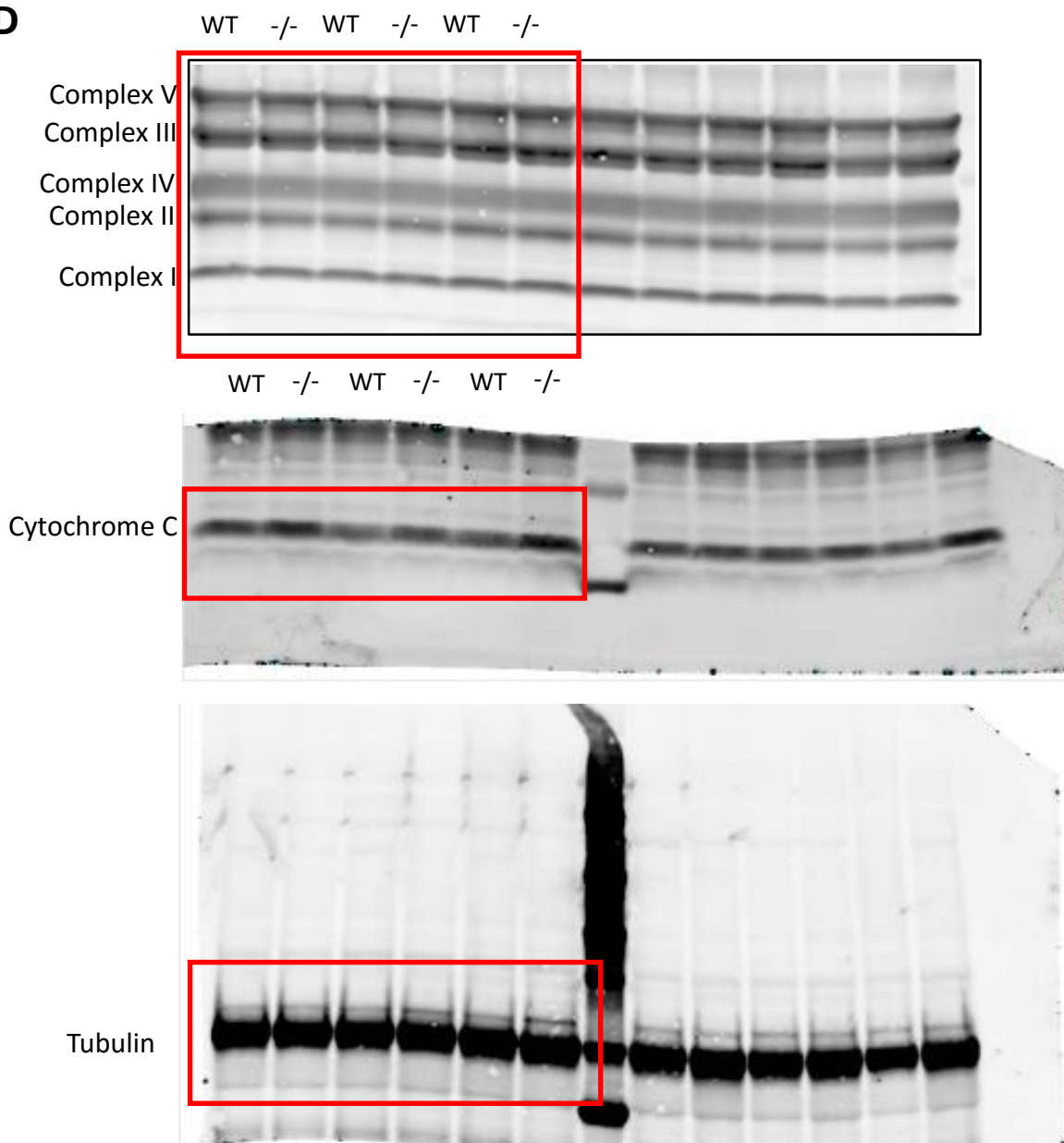

**E**

E2  
E3bp

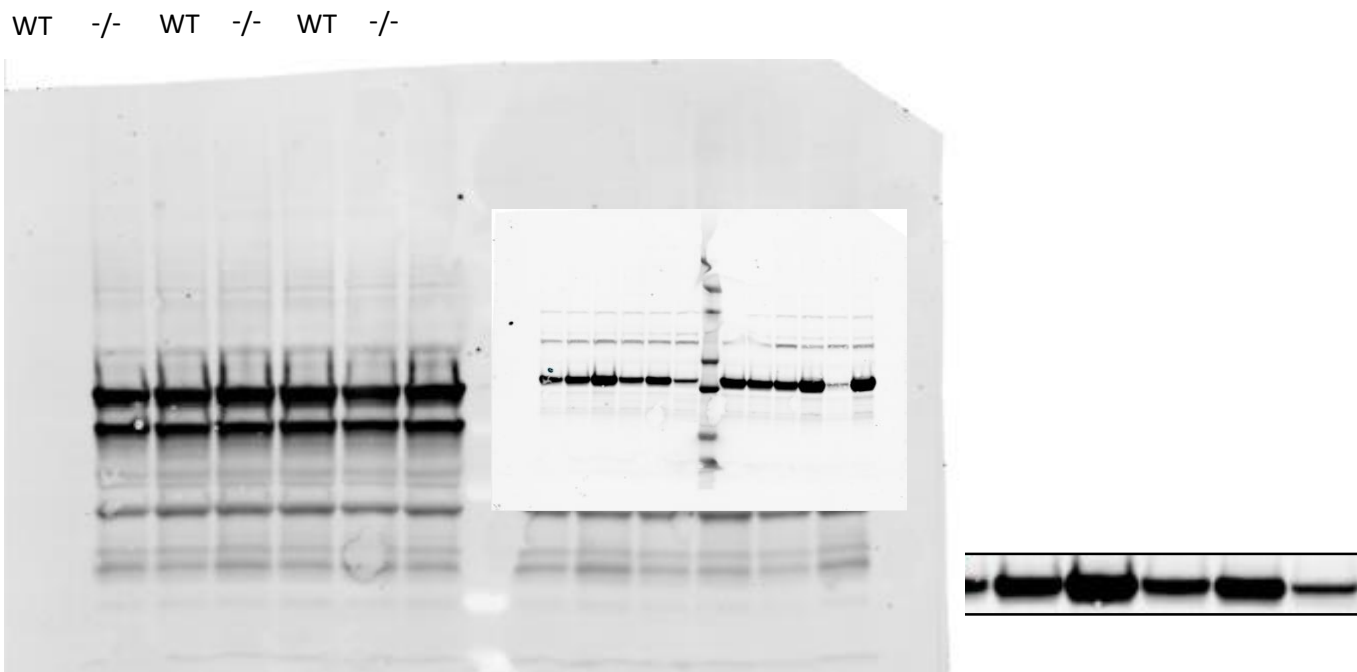

Figure 4

E

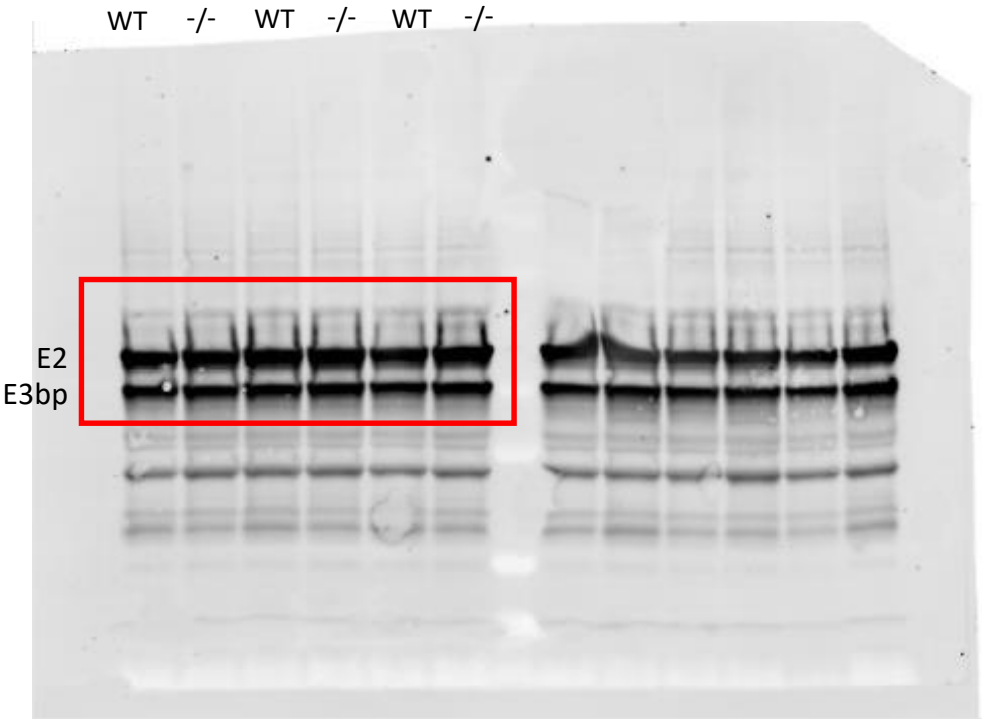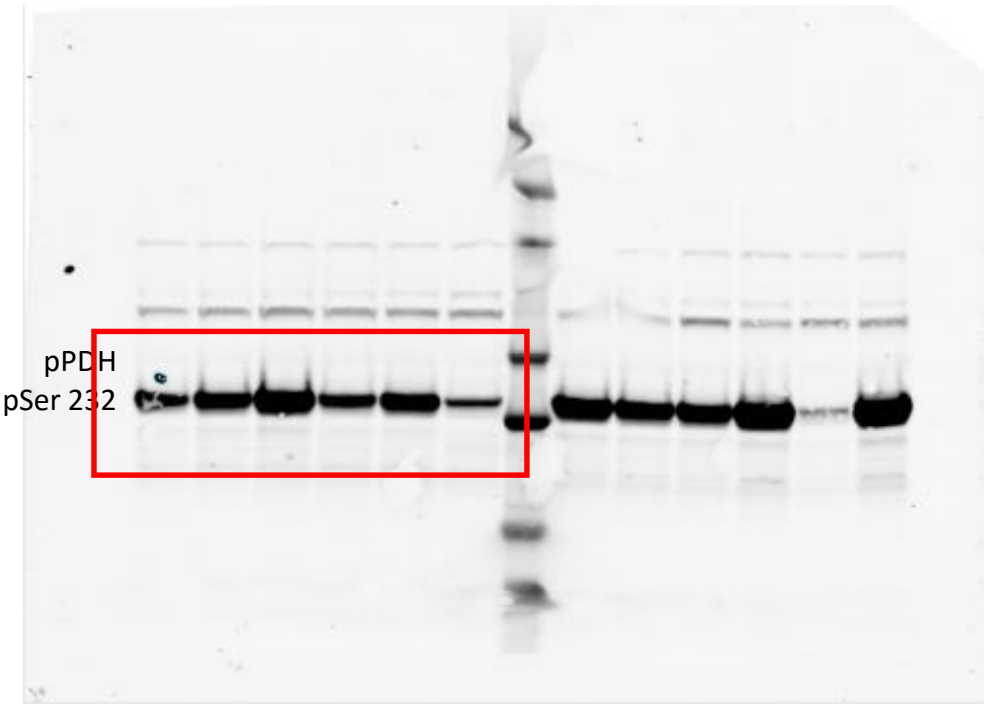

**Figure 5**

**B**

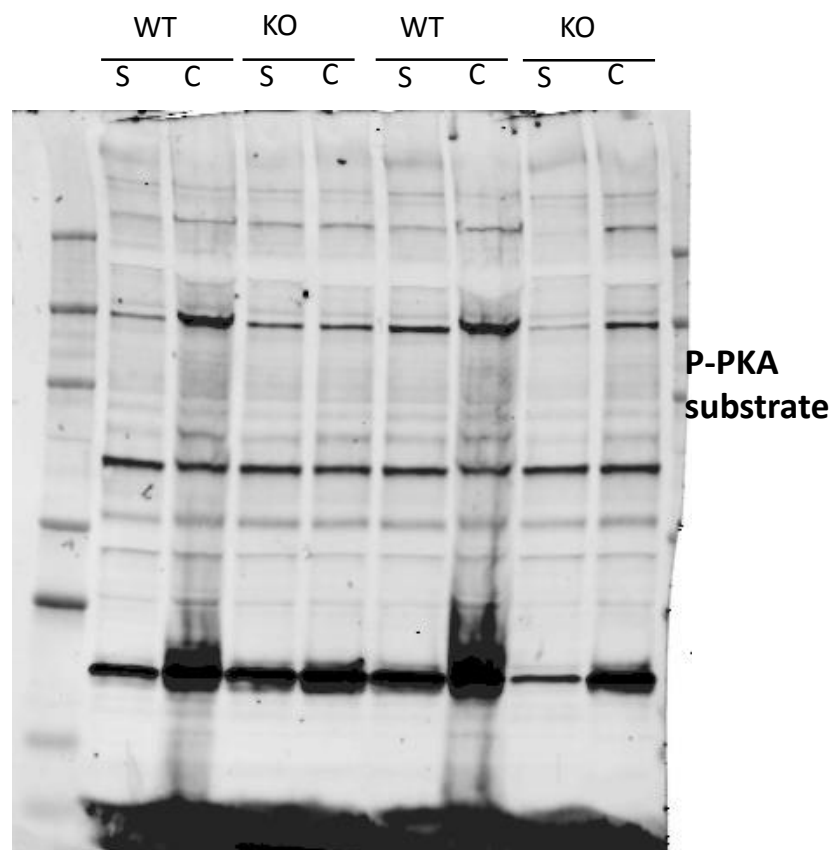

Supplement: Supplemental data [file jciinsight-6-134340-s161.pdf]
